# Effects of microbiome-based interventions on neurodegenerative diseases: a systematic review and meta-analysis

**DOI:** 10.1038/s41598-024-59250-w

**Published:** 2024-04-26

**Authors:** Zara Siu Wa Chui, Lily Man Lee Chan, Esther Wan Hei Zhang, Suisha Liang, Edmond Pui Hang Choi, Kris Yuet Wan Lok, Hein Min Tun, Jojo Yan Yan Kwok

**Affiliations:** 1 School of Biomedical Sciences, Li Ka Shing Faculty of Medicine, The University of Hong Kong, Pokfulam, Hong Kong SAR China; 2 School of Nursing, Li Ka Shing Faculty of Medicine, The University of Hong Kong, Pokfulam, Hong Kong SAR China; 3 Faculty of Medicine, The Chinese University of Hong Kong, Shatin, Hong Kong SAR China; 4 HKU-Pasteur Research Pole, School of Public Health, Li Ka Shing Faculty of Medicine, The University of Hong Kong, Pokfulam, Hong Kong SAR China; 5 The Jockey Club School of Public Health and Primary Care, Faculty of Medicine, The Chinese University of Hong Kong, Hong Kong SAR, China; 6 Microbiota I-Center (MagIC), Hong Kong SAR, China; 7 Li Ka Shing Institute of Health Sciences, Faculty of Medicine, The Chinese University of Hong Kong, Hong Kong SAR, China; 8 Centre on Behavioral Health, The University of Hong Kong, Pokfulam, Hong Kong SAR, China

**Keywords:** Neurodegenerative disease, Gut microbiome, Multiple sclerosis, Alzheimer’s disease, Parkinson’s disease, Microbiome modulating interventions, Neurodegenerative diseases, Microbiome

## Abstract

Neurodegenerative diseases (NDDs) are characterized by neuronal damage and progressive loss of neuron function. Microbiome-based interventions, such as dietary interventions, biotics, and fecal microbiome transplant, have been proposed as a novel approach to managing symptoms and modulating disease progression. Emerging clinical trials have investigated the efficacy of interventions modulating the GM in alleviating or reversing disease progression, yet no comprehensive synthesis have been done. A systematic review of the literature was therefore conducted to investigate the efficacy of microbiome-modulating methods. The search yielded 4051 articles, with 15 clinical trials included. The overall risk of bias was moderate in most studies. Most microbiome-modulating interventions changed the GM composition. Despite inconsistent changes in GM composition, the meta-analysis showed that microbiome-modulating interventions improved disease burden (SMD, − 0.57; 95% CI − 0.93 to − 0.21; I^2^ = 42%; *P* = 0.002) with a qualitative trend of improvement in constipation. However, current studies have high methodological heterogeneity and small sample sizes, requiring more well-designed and controlled studies to elucidate the complex linkage between microbiome, microbiome-modulating interventions, and NDDs.

## Introduction

Neurodegenerative diseases (NDDs) are a diverse spectrum of disorders characterized by the progressive loss of neurons and deterioration in the central or peripheral nervous system, resulting in long-term motor and nonmotor impairments^1^. NDDs include Parkinson’s disease (PD), Alzheimer’s disease (AD), frontotemporal dementia and its variants, amyotrophic lateral sclerosis (ALS), and multiple sclerosis (MS). As the population ages, the incidence rate and prevalence of NDDs increase modestly, as demonstrated by an incidence estimated annual percentage changes of 0.52 for PD and 0.13 in men and 0.06 in women for AD^2,3^. Affecting millions of people worldwide, NDDs are a major public health concern; yet, despite decades of research effort, no effective treatments for curing or reversing their progression have been realized^3^. The exact pathophysiology of NDDs is also not fully elucidated owing to the heterogeneity and complexity of these diseases^4–6^. However, emerging evidence suggests that the gut microbiome (GM), the collection of microorganisms that inhabit the gastrointestinal (GI) tract, may play a role in modulating the risk and severity of NDDs.

GM, often called the second brain, harbors nearly 100 trillion bacteria, yeast, and other microorganisms, functioning symbiotically in day-to-day activities^7^. Host genetics, lifestyle, and environmental factors, such as diet, chemical exposure, infection, and host comorbidity, shape GM through the modulation of gut motility and secretion, which in turn affects various aspects of the host physiology, including immunomodulation, metabolic activity, and neuronal development and function. The connection between GM and metabolic and immune-related diseases is well established^8^. For example, obesity, as a complex metabolic disorder, is associated with decreased diversity and richness and altered composition in GM^9^. Wells et al. also identified that *Prevotella* correlates with the genetic risk and anticitrullinated protein antibody level of rheumatoid arthritis, suggesting the role of *Prevotella* as a potential mediator in disease progression^10^.

A growing body of evidence has suggested that GM also communicates bidirectionally via multiple pathways, which collectively is described by the gut–brain axis. The brain communicates with the gut through neuronal and hormonal pathways, including the hypothalamic pituitary adrenal axis (HPA) and sympathoadrenal axis^11^. The vagal nerves relay most signals from the brain to the gut^12^ and coordinate stress and anti-inflammatory activities with HPA to regulate gut motility, intestinal permeability, and mucosal immune activity^13^. At the same time, GM can affect the brain by producing and releasing various molecules, such as metabolites, neurotransmitters, and cytokines; these molecules can reach the brain through multiple pathways and may be a key modulator in NDDs^14^.

Disruption of GM balance caused by host and environmental factors may lead to diseases or disorders^15^. Romano et al. performed a meta-analysis of 21 case–control studies to compare the GM composition of 1083 PD patients and 1213 healthy controls and revealed a lower abundance of *Prevotellaceae* and *Lachnospiraceae* families and higher abundance of *Enterobacteriaceae* and *Akkermansiaceae* families in patients than in controls^16^. Similarly, patterns of dysbiosis in other NDDs, including AD, MS, and ALS, have been reported in recent systematic review and meta-analysis^17–19^. Sampson et al. reported the requisite involvement of gut microbiota to elicit synucleinopathies in a PD model using wild-type and Thy1-α-synuclein genotype mice, in which the germ-free Thy1-α-synuclein genotype demonstrated limited motor and GI dysfunction compared with specific pathogen-free counterparts^20^. These findings suggest that GM may play a key role in the pathophysiology of NDDs, and modulating GM may be a potential strategy for preventing or treating NDDs.

However, many challenges and limitations remain in this research field. For example, most animal studies rely on germ-free or genetic models of NDDs, which may not fully recapitulate the human disease phenotypes or etiologies^21^. Standardized methods for assessing and manipulating GM across different studies are also lacking. Meanwhile, human studies, are mostly observational and cross-sectional, which cannot establish causality or directionality between GM and NDDs^22^.

In the recent decade, clinical trials have been conducted to investigate the efficacy of interventions modulating GM in alleviating or reversing disease progression. Yet, comprehensive synthesis of the available evidence in understanding microbiome-modulating methods is lacking. Therefore, this systematic review aims to summarize and critically appraise the current evidence regarding the effects of microbiome-modulating interventions on NDD-related clinical outcomes and to discuss the translatability and implementation potentials for future research and clinical application.

## Methods

### Inclusion criteria and search strategy

This protocol-based systematic review (PROSPERO ID: CRD42023437490) was conducted and reported in accordance with the Cochrane Handbook for Systematic Reviews of Interventions^23^ and the Preferred Reporting Items for Systematic Reviews and Meta-Analyses statement^24^. Studies were selected in accordance with predefined inclusion and exclusion criteria. Randomized controlled trials (RCTs), quasi-experimental studies, single-arm studies, and pilot studies with microbiome-modulating intervention were included if studies (1) were conducted in adults (age > 18 years) with a diagnosis of NDDs, such as AD, PD, MS, and ALS, and (2) reported any microbiome outcomes. Microbiome-modulating intervention is defined as any treatment or intervention that alters the composition, diversity, or functionality of GM. The intervention can be, but not limited to, changes in diet or lifestyle, use of biotics, fecal microbiota transplantation (FMT), or other medications. The methods of microbiome analysis are not restricted, which may include, but not limited to, 16S ribosomal (r) RNA sequencing, shotgun metagenomic sequencing, and fluorescence in situ hybridization. Studies were excluded if they were not published in English.

PubMed, Ovid-Embase, and Web of Science were searched from inception to January 11, 2023. The search strategy was summarized as follows: [neurodegenerative diseases] AND [microbiome assessment] AND [microbiome-modulating methods: (diet) OR (supplement or biotics) OR (FMT)]. Online Resource 1 presents the full search strategy. Duplicate records were removed with EndNote and manually.

### Data extraction and quality assessment

Two independent reviewers (ZSW Chui and EWH Zhang) extracted data from each trial using a pre-specific, standardized form and evaluated the risk of bias via the Cochrane Risk-of-Bias Tool 2 for RCTs^25^ and the Risk Of Bias In Nonrandomized Studies of Interventions (ROBINS-I) for non-RCTs^26^. Discrepancies were identified and resolved by consensus with a third reviewer (LML Chan) and the supervisor (JYY Kwok).

### Synthesis

A narrative synthesis was conducted for all trials to describe study design, country and setting of study, characteristics of participants and interventions, assessment time points, microbiota sequencing technique, main microbiota, and clinical outcomes (Table 1). Meta-analyses were performed among microbiota and clinical outcomes if they were reported by at least three studies. In view of wide variations in instruments used between trials to assess the primary outcomes, pooled effects were summarized as standardized mean differences (SMDs). SMD is a summary statistic used to report intervention effects in standardized units, rather than the original units of measurement for each scale. The total sample size, mean with standard deviation (SD) or median, and interquartile range of disease progression pre- and post-intervention were extracted to calculate SMD and SD. Twenty-five individual study results were corrected for directionality when appropriate. Considering the substantial variations in microbiome-modulating interventions and study design, we utilized a random-effect model to conduct the analysis using RevMan 5.4^27,28^. All significance tests were 2-tailed, and *P* value of < 0.05 was considered statistically significant. The heterogeneity among studies was assessed using I^2^ statistic.

**Table 1:** Summary of clinical studies of microbiome modulating interventions for neurodegenerative diseases.

Study	Study Design	NDD	Interventions	Study duration and assessment timepoint	Study population	Baseline characteristics	Main microbiota outcome	Main clinical outcome
Al et al^22^	Pilot RCT	MS	[1] early rectal enema: FMT per month for six months followed by six months observation, [2] late rectal enema: six months monitoring followed by FMT per month for six months. [1] and [2] randomized to receive FMTs from donor 1 or donor 2	12 months, monthly assessment Terminated early due to unexpected death of primary investigators Assess peripheral blood cytokine concentrations, gut microbiota composition, intestinal permeability, and safety measured by EDSS and MRI activity	[1]: N = 4, [2]: N = 5	Age: 44 ± 8.2, average age of diagnosis: 32.1 ± 8.5, Disease duration: 14.6 ± 6.8	No significant change in alpha and beta diversity Donor specific alterations that those receive from donor 1 has enriched taxa and functional output *Hungatella hathewayi, metsC, menC, tauB,* and ubiquinol biosynthesis whereas those receive from donor 2 has enrinched *phscolarctobacterium succinatutens* and *hasA*	**Functional/nonfunctional outcomes:** No grade 3 or 4 adverse events, no sig changes in EDSS and new MRI activity **Inflammatory tone:** cytokines result was underpowered. No sig changes in the levels of any cytokines measured post FMT or compared to healthy controls **Intestinal permeability:** normalized post-FMT
Barone et al^29^	Single-arm study	MS	1-week High-Impact Multidimensional Rehabilitation: (a) Tailor-made neuromotor rehabilitation session; (b) Recommended diet mainly based on the Mediterranean diet principles; (c) Designed sailing course proposed with equipped single- and double-seated monohulls; (d) Mindfulness	1 week, prior to and post intervention (day 0 and day 7) Assess clinical and nutritional variables (by 6MWT, MFIS-5, FFQ), serum/blood analysis, cortisol detection, and gut microbiota analysis	N = 14	Age: 49.93 ± 9.08, male: 50%, Disease duration: 19.25 ± 5.4, EDSS: 5.3 ± 1.66	No differences in intra- and inter-sample variability Partial recovery of dysbiosis: reduced phylum *Actinobacteria*, family C*oriobacteriaceae* and peptostreptococcaceae, depleted proinflammatory genus *Ruminococcus*, increased *Bacteroidaceae* and *barnesiellaceae* SCFA producers: *Blautia* remained stable, and increased *Coprococcus*, *Bacteroides*, and *Oscillospira*, but other SCFA producers reduced. Decreased *Eggerthella*	**Functional/nonfunctional outcomes:** Improved MFIS-5, 6MWT, 6MWT-dynamic index, adherence to anti-inflammatory diet **Inflammatory tone:** Decreased CD4 + /IFN-γ + , Th1, CD4 + /ROR-γ + , CD4 + /IL-17 + , and Th17 and serum LPS. Increased I-FABP No significant difference in serum cortisol
Tankou et al^30,31^	Single-arm study	MS	Probiotics: LBS supplementation twice daily for two months LBS: contain L. paracasei DSM 24,734, L.plantarum DSM 24,730, L. acidophilus DSM 24,735, L. delbruckei subspecies bulgaricus DSM 24,734, B. longum DSM 24,736, B. infantis DSM24737, B. breve DSM 24,732, and Streptococcus thermophilus DSM 24,731	5 months, prior to, at discontinuation of therapy (t: 2 months), and 3 months thereafter for collecting blood and stool specimens Assess microbiome, stool metabolomics, PBMCs, and immune gene expression	N = 22([HC]:N = 13, [MS]: N = 9)	[HC] Age: 35 ± 14, male: 38.5%, BMI: 25.8 ± 4.1; [MS] Age: 50 ± 10, male: 44.4%, BMI: 31.1 ± 5.6, EDSS: 1.4 ± 0.9	Decreased alpha diversity in [HC] but not [MS]. Beta diversity changed significantly and shifted back to baseline following discontinuation in both groups *Veillonellaceae* and *Collinsela* increased in [HC], decreased *Akkermansia, Blautia, Dorea, and B. adolescentis* in both groups Decreased KEGG pathways in both groups: Metabolism, Cellular Processes, Environmental Information and processing, Organismal System, and Methane metabolism	**Stool metabolites**: increased uracil, AMP, hypoxanthine, xanthine in [HC] after LBS supplementation, increased 2-oxoglutarate after discontinuation in [HC]. Decreased 3-hydroxyvalerate in [MS] after LBS supplementation, increased 3-Methyl-oxovalerate, citrate, nicotinate, alpha-ketoisovalerate after discontinuation in [MS] **PBMC** after LBS supplementation: no sig change in relative frequencies of CD4^+^CD127^low^CD25^high^ and decrease trend in Th1 and Th17 in both groups, trend of increase in effector memory CD8 after supplementation in [MS], trend of decrease in LAP^+^ T regs in [HC], PBMS after discontinuation: decreased CD4^+^IL^-^10^+^ and CD39^+^CD127^low^CD25^high^ T regs **Monocytes:** decreased frequency of intermediate monocytes and decreased MFI of HLA-DR on myeloid derived dendritic cells in [MS], trend of decreased of inflammatory monocytes and MFI of CD80 on classical monocytes in [HC] after LBS supplementation **Gene expression:** increased *IL-10RA, LILRB2*, *CYBB*, and decreased *MALT1* and *LGALS3* after LBS supplementation in [MS]. *CYBB* remain decreased after discontinuation. Decreased *HLA.DQA1, HLA.DPA1* and *IL6ST* in [HC] monocytes after LBS supplementation, which *HLA.DPA1* and *HLADPB1* increased but *IL6ST* remain decreased after discontinuation. Decreased *PTNP2* after discontinuation in [HC] **Correlations in [HC]:** negative correlation between CD80 MFI and 5 *Streptococcus,* between *HLA.DPA1* and *HLA.DPB1* expression, between *Lactobacillus* and *Bifidobacterium* NR847, and between hypoxanthine and *HLA.DPA1*, positive correlation between *Bifidobacterium* OTU 1,142,029 and MFI CD80 and IL6ST, between hypoxanthine and PTPN2, **Correlations in [MS]:** negative correlation between 2 *Lactobacillus* and MFI HA-DR, between *CYBB* and 2 *Streptococcus*, *HLA.DPB1,* positive correlation between OTU 1,142,029 and *CYBB*, trend of positive correlation between *Streptococcus* NROTU0 and LILRB2
Cignarella et al^32^	Single-blind RCT	MS	[I] alternate-day fasting for 15 days with corticosteroid treatment; [C]: corticosteroid treatment and regular diet for 15 days Corticosteroid treatment: 10-day oral steroids (dexamethasone or prednisone) or 3-day IV methyl prednisolone followed by 8-day oral corticosteroid taper	15 days; prior to and post intervention (day 1 visit and day 15 visit) Assess white blood cells, Treg functions, gut microbiome composition, general physical and neurological assessments such as EDSS, MSFC, SDMT, MS-QoL	[I]: N = 8, [C]: N = 9	[I] Age: 40 ± 12, Male: 37.5%, BMI: 30.2 ± 5.8, Waist circumference: 96.9 ± 10.2, Disease duration: 7.8 ± 6.4, EDSS: 3.7 (3.2–4); [C] Age: 42 ± 8.2, Male: 12.5%, BMI: 31.2 ± 6.4, Waist circumference: 106.6 ± 13.7, Disease duration: 8.5 ± 8.1, EDSS: 3.7(2.7–5.2)	[I]: decreased phylum *Actinobacteria, Bacteroidetes and Verrucomicrobia,* increased *Firmicutes,* SCFA producers: increasing trend of *Faecalibacterium, Lachnospiracea_incertae_sedis and Blautia* Magnitude of changes in [C] was much lower but no bacteria were significantly different at day 15 between two groups	**Functional/nonfunctional outcomes:** improved EDSS without any significant difference in the degree of amelioration, no difference in MSFC and SDMT **Adipokines and metabolites:** Increased serum adiponectin but no change in beta-hydroxybutyrate, [I]: Decreased BMI to significantly different from [C], Reduced serum leptin **Immune cells** [I]&[C]: Increased white blood cells mainly driven by neutrophils
Di Gioia et al.^33^	prospective longitudinal study followed by pilot RCT	ALS	6-month probiotics including Streptococcus thermophilus ST10–DSM, Lactobacillus fermentum LF10–DSM 19,187, and Lactobacillus delbrueckii sub sp. delbrueckii LDD01–DSM 22,106, Lactobacillus plantarum LP01–LMG *P*-21021, and Lactobacillus salivarius LS03–DSM 22,776	7 months ([I] 1 month observation + 6 months supplement, [C] 3 months placebo + 3 months supplement) Assessed monthly for ALSFRS-R, FVC%, BMI, dietary habits Collected stool samples at baseline (T0), after three months (T1) and after 6 months (T2)	N = 50 [I] N = 25, [C] N = 25	[I] Age 60.36 ± 10.86, BMI: 24.82 ± 3.95, FVC%: 81.48 ± 18.28; [C] Age: 59.64 ± 8.12, BMI: 24.86 ± 3.97, FVC%: 83.9 ± 18.46. Lower Clostridium and yeast concentration, higher E. Coli in ALS patients compared to HC	Intervention did not bring the gut microbiota biodiversity of ALS patients closer to that of controls. Significant decrease in the number of OTO observed during follow-up At T1, lower bacterial count in [C] with respect to [I] At T2, significant reduction of yeast concentration in [I] No sig difference in total bacteria counts and single bacterial group, except increased *E. coli* in [C]. considerably higher *Rikenellaceae* in [I]	**Functional/nonfunctional outcomes:** No improvement on disease progression, BMI, ALSFRS-R, Bubar ALSFRS-R, Delta ALSFRS-R: did not differ; FVC% decreased; FVC related to microbiota regardless of treatment and time
Becker et al^34^	Non-RCT	PD	[1]: PD patients receive 5 g of resistance starch twice a day for 8 weeks. [2] PD patients were told to follow habitual diet (usual care control). [3] Healthy controls receive 5 g of resistance starch twice a day for 8 weeks	8 weeks; prior to, at 4 weeks, and at 8 weeks Clinical assessments include CSS, BDI, NMSQ, CGI. Assess microbiota composition, fecal SCFA and calprotectin concentration	[1]: N = 32, [2]: N = 25, [3]: N = 30	[1] age: 64.5 (42–84), male: 56.25%, Disease duration: 9.25 (0.58–24), UPDRS score: 35(4–74), MMST: 29(23–30) [2]age: 66 (47–80), male: 52%, Disease duration: 9.25 (1.83–22.1), UPDRS score: 30(3–69), MMST: 29(25–30); [3] age: 61.5 (40–76), male: 40%	No significant intervention-associated changes with regard to alpha- and beta-diversity and distinct taxa [1]: significant differences in metagenomic signature derived from Rhodococcus	**Functional/nonfunctional outcomes:** [1] improved NMSQ, BQI [2, 3]: no changes in NMSQ and BQI [1–3]: no change in CSS **Fecal SCFAs:** [1]: significant increase in absolute and relative fecal butyrate concentration, no changes in other SCFAs. [2, 3]: no changes in SCFAs
Kuai et al^35^	Single-arm study	PD	One-time FMT	12 weeks; before, at 4,8, and 12 weeks after FMT Assess H-Y grade, UPDRS, NMSS, PAC-QOL, Wexner constipation score, microbiota composition, LHBT,	N = 11	age: 62.45 ± 13.08, Male: 63.63%, disease duration 7.18 ± 3.25 years, H-Y Grade 2.27 (range 1–3), UPDRS Score 11.36 (range 10–19), NMSS 22.36 (range:14–32). PACQOL score 102.55 (range 93–108) , Wexne rconstipation score 11.63 (range 5–12)	Significantly increased species diversity and the pattern of the richness, which becomes insignificantly different from HCs Family *Coriobacteriaceae, Erysipelotrichaceae, Lachnospiraceae,* genus *Collinsella, Eubacterium_hallii_group, Ruminococcus_1, Dorea, Blautia, Romboutsia* became dominant Significant and gradual decrease in *Bacteroides and Escherichia-Shigella.* Increased *Faecalibacterium* at 4 and 12 weeks post-FMT, increased *Blautia* at 8 and 12 weeks post-FMT	**Functional/nonfunctional outcomes:** H-Y scores decreased, UPDRS, NMSQ, PAC-QOL, Wexner constipation score increased, **small intestinal bacterial overgrowth diagnosis**: significantly decreased, HCY expression increased
Hegelmaier et al^36^	Non-RCT	PD	[1]: idiopathic PD receiving 8-day enema and 14-day ovo-lacto vegetarian diet; [2]: idiopathic PD receiving 14-day ovo-lacto vegetarian diet only	1 year; two days prior to treatment, post-treatment (day 14), at one-year follow-up Assess UPDRS-III, levodopa dosage, Bristol stool scale	[1]: N = 10; [2]: N = 6;	Age: 64 ± 5.4, Male: 37%, disease duration: 8.6 ± 4.1, BMI: 26.7 ± 4	No change in alpha diversit [1]: significant reduction of *Clostridiaceae*	**Functional/nonfunctional outcomes:** UPDRS-III significantly improved (more significant in [1] than [2]), levodopa-equivalent daily dose decreased at one-year follow-up Correlation between abundance of *Ruminococcaceae* and UPDRS-III
Rusch et al.^37^	Single-arm study	PD	5-week Mediterranean diet	7 weeks (2-week observation and 5-week intervention); Daily questionnaire on stool frequency, Weekly GI symptoms questionnaire with Bristol Stool Scale Visit 1—last day of the 2-weeks baseline observation: MDS-UPDRS, MoCA, stool and urine sampling for microbiome and intestinal permeability analysis post-intervention: stool and urine sampling	N = 8	age: 71.4 ± 2.6, male: 62.5%, BMI: 26.7 ± 1.4, MDS-UPDRS: 54. + ± 9.9, MoCA: 26.6 ± 1.0	alpha- and beta- diversity unchanged 98% similar OTUs: *Desulovibrionaceae* and *Bilophila* reduced, *Roseburea* increased at week 5 95% similar OTUs: *Clostrium bolteae, Ruminococous, Blautia, Dorea and Lachnospiraceae* decreased	**GI outcomes:** Decreased body weight, significantly lower GSRS constipation and indigestion score, unchanged number of bowel movement and BSS; **Intestinal permeability:** significant increased 0-5 h lactose, 0-5 h erythriol, and 5-24 h sucralose excretion yet urinary excretion ratios of markers of intestinal permeability did not differ
Sun et al^38^	RCT	PD	[I] 2 g of Probio-M8 daily plus conventional drugs (Benserazide); [C] 2 g of placebo (maltodextrin) plus conventional drug (Benserazide)	3 months; T0 = baseline, T1 = 1 month, and T2 = 3 month Assess UPDRS-III, MMSE, HAMA, HAMD-17, PDSS, VAS, ADL, PAC-QoL, Bristol Stool Score, self-adminstered questionnaire regarding clinical and GI-related issues, microbiome composition, metabolic modules and metabolites, fecal SCFAs	N = 100 [I]: N = 50, [C]: N = 50	Age: 67 ± 7.05, Male: 67%, UPDRSIII: 16.869 ± 8.43	No signficant change in alpha- and beta- diversity [I]: significant increase in *B. animalis, Ruminococcaceae, Lachnospira, and Butyricimonas*; less *Lactobacillus fermentum* and *K. oxytoca*, increased diversity of SGBs in tryptophan degradation, GABA, SCFAs, and secondary bile acid synthesis; [C]: more diverse SGBs participating in vitamin K2 synthesis, tryptophan synthesis, and inositol degradation; [I] vs [C]: significantly different abundance in *Butyricimonas sp* at T1, significantly difference in 28 SGBs	**Functional/nonfunctional outcomes:** [I]&[C]: improved UPDRSIII, MMSE, HAMA, HAMD-17, more significant improvement in [I]. Improvement in UPDRS-III was only significant at T2 in [C]. [I]: improved PDSS and higher possibility of continuing medication **GI outcomes:** [I] improved GI-related symptoms such as times of spontaneous defecation and completed defecation per week **Metabolites and SCFAs: [I]** higher serum acetate at T1 and T2. Significantly higher dopamine, and significantly lower glutamine and tryptophan concentration in [I] than [C] a T1
Hong et al^39^	Open-label single-arm study	PD	550 mg of rifaximin twice per day for 7 days	6 months; 1 week intervention with 6-month follow up; Baseline: gut microbiome, clinical performance, blood samples; immediately post intervention: gut microbiome only, 6 months post intervention: motor performance and blood samples	N = 13	Age: 61.59 ± 5.34, Male: 54%, disease duration: 1.77 ± 1.74, UPDRSIII: 13.69 ± 8.75	No significant change in overall relative abundance, alpha- and beta- diversity Increased abundance of *Flavonifractor,* but no other bacterial genera in the patients	**Functional/nonfunctional outcomes:** UPDRS decreased (baseline: 13.69 ± 8.75, 6-month post rifaximin: 12.31 ± 9.21) **Inflammatory tone:** increased trend of serum anti-inflammatory cytokine, significant increase in IL-10 only. Significant negative correlation between baseline IL-1α level and changes in the levels of the proinflammatory cytokines IL-1α, IL-1β, IFN- γ, TNF- α
Kountouras et al.^40^	Non-RCT	AD	[1]: Hp positive AD patients, a triple Helicobacter pylori eradication regimen (omeprazole, clarithromycin andamoxicillin) [2]: Hp positive AD patients, unsuccessful or denied eradication [3]: Hp negative AD patients Hp-eradication therapy: 1-week omeprazole (20 mg bid), clarithromycin (500 mg bid), and amoxycillin (1 g bid), followed by omeprazole 20 mg once daily for 1 month	5-week Hp eradication therapy with 2-year follow-up; prior to intervention, at least 8 weeks after cessation of therapy, one and two years after therapy Assess MMSE, CAMCOG, FRSSD, upper GI endoscopy, Hp detection	[1]: N = 28, [2]: N = 28, of which 5 had unsuccessful eradication, [3]: N = 5	Age: 65.0 ± 6.9, Male: 36%, H. pylori positive: 88%, serum anti H. pylori IgG (U/ml): 34.0 ± 40.1. late enrolment patients belonging to group B (N = 16) age: 74 ± 6.83, Male: 32.25%, H.pylori positive: 100%	Successful Hp eradication rate = 84.85%	**Functional/nonfunctional outcomes:** MMSE, CAMCOG and FRSSD are significant improved in [1], deteriorated in [2], unchanged in [3]
Nagpal, R., et al.^41^	double-blind, cross-over, single-center pilot RCT	AD	Modified Mediterranean-ketogenic diet (MMKD)/ American Heart Association Diet (AHAD),	18 weeks (6 weeks intervention followed by 6 weeks of washout period, and then 6 weeks intervention with the second diet); before diet 1, at the end of diet 1, before diet 2(no LP), at the end of diet 2 Collect blood and stool samples at the four visits. Lumbar puncture was also conducted except prior to diet 2	[MCI] N = 11, [HC] N = 6	Age: 64.6 ± 6.4, Male: 29.4%, APOE ε4 (E4) genotype: 35.3%	Diet do not show strong effect on overall alpha and beta diversity indices Post MMKD: The abundance of family *Bifidobacteriaceae,* genus *Bifidobacterium* decreased, which is more prominent in [MCI]. Abundance of family *Enterobacteriaceae,* genera *Akkermansia, Slackia, Christensenellaceae and Erysipelotriaceae* increased. Genera *lachnobacterium* decreased Abundance of KEGG pathways associated with Alzheimer;s disease, type-1diabetes, type-2 diabetes, and bacterial toxin is decreased Post AHAD: increased *Mollicutes,* abundance of gene families associated with carbohydrate digestion and absorption is slightly increased	**CSF biomarkers:** *Firmicutes* positively correlates with tau-p181, *Proteobacteria* negatively correlated with Ab42 mainly in [HC] but positively correlated with Ab42/Ab40 ratio in [MCI]. *Enterobacteriaceae* positively correlates with tau-p181 and tau-p181/Ab42 ratio Lactate level is positively correlated with tau-p181 post AHAD in [HC] **Fecal metabolites/SCFAs:** overall both reduced lactate and increased propionate. MMKD slightly reduces faecal lactate and acetate while increasing propionate and butyrate. AHAD increases acetate and propionate while reducing butyrate
Leblhuber et al.^42^	single-arm study	AD	Aqueous suspensions of the probiotic Omnibiotic Stress Repair consisted of Lactobacillus casei W56, Lactococcus lactis W19, Lactobacillus acidophilus W22, Bifidobacterium lactis W52, Lactobacillus paracasei W20, Lactobacillus plantarum W62, Bifidobacterium lactis W51, Bifidobacterium bifidum W23 and Lactobacillus salivarius W24	28 days, before and after probiotic supplementation (day 0 and 28) Measure fecal inflammation markers calprotectin, α1-antitrypsin, zoulin, neopterin Vit D, BDNF, aromatic amino acid. Performed routine lab test, assessed MMSE and CDT	N = 20	Age: 76.7 ± 9.6, Male: 45%, MMSE: 18.5 ± 7.7, CDT: 4.3 ± 2.7, serum concentration of CRP: 1.6 ± 2.3	increase in *Faecalibacterium prausnitzii* compared to baseline	**Functional/nonfunctional outcomes:** cognitive parameters unchanged (MMSE and CDT) **Inflammatory tone:** Decreased faecal zonulin concentrations and increased serum kynurenine and nitrite concentration. Delta values (before—after) of neopterin and the kynurenine to tryptophan ratios (Kyn/Trp) correlated significantly (*p* < 0.05)

*ALSFRS-R* Amyotrophic Lateral Sclerosis Functional Rating Scale-Revised, *BDI* Beck Depression Inventory, *BMI* Body Mass Index, *BQI* Bowel Quality of Life Index, *CAMCOG* Cambridge Cognitive Examination, *CDT* clock drawing test, *CGI* Clinical Global Impression, *CSS* Constipation Scoring System, *EDSS* Expanded Disability Status Scale, *FFQ* Food Frequency Questionnaire, *FRSSD* Functional Rating Scale for Symptoms of Dysphagia, *FVC* Forced Vital Capacity, *GSRS* Gastrointestinal Symptom Rating Scale, *HAMA* Hamilton Anxiety Rating Scale, *HAMD-17* Hamilton Depression Rating Scale—17 items, *HCY* Homocysteine , *H-Y* Hoehn and Yahr scale, *LHBT* Lactose Hydrogen Breath Test, *MDS-UPDRS* Movement Disorder Society, Unified Parkinson's Disease Rating Scale, *MFI* Multidimensional Fatigue Inventory, *MFIS-5* Modified Fatigue Impact Scale—5 items, *MMSE* Mini-mental state examination, *MRI* Magnetic Resonance Imaging, *MSFC* Multiple Sclerosis Functional Composite, *MS-QoL* Multiple Sclerosis-Quality of Life, *NMSQ* Non-Motor Symptoms Questionnaire, *OUT* Operational Taxonomic Unit, *PACQOL* Parkinson's Disease Questionnaire on Quality of Life, *PBMC* Peripheral blood mononuclear cell, *PDSS* Parkinson's Disease Sleep Scale, *SDMT* Symbol Digit Modalities Test, *UPDRS* Unified Parkinson's Disease Rating Scale, *6MWT* Six-Minute Walk Test.

## Results

### Study selection

**Figure 1:**
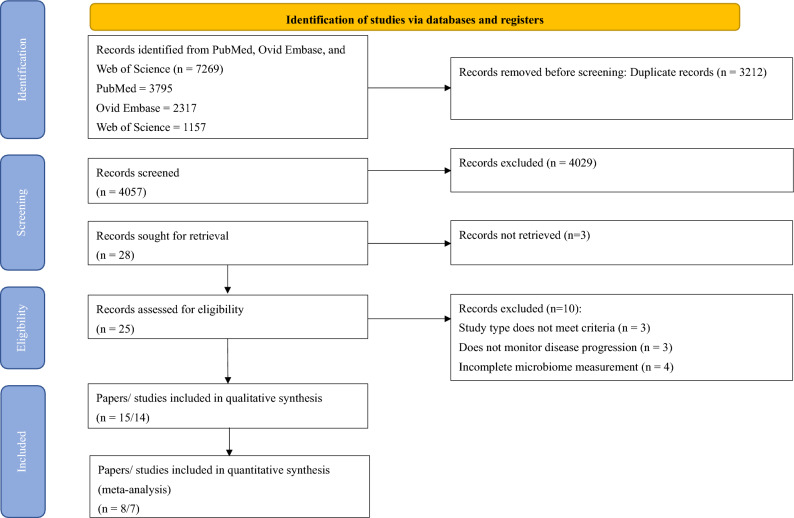
Preferred reporting items for systematic reviews and meta-analyses (PRISMA) flowchart.

The PRIMSA 2020 flow diagram (Fig. 1) shows the flow of the study selection process. A total of 7269 unique records identified from the search, of which 25 were deemed eligible for full review. Fourteen trials were included for qualitative synthesis. Seven trials were included in the meta-analyses.

### Study characteristics

Table 1 shows a systematic presentation of information regarding the study characteristics. Fifteen articles comprised 14 trials that involved 445 patients: 5 trials/6 articles on sclerosis (n = 112), 6 trials on PD (n = 235), and 3 trials on AD and related disorders (n = 98). Of the 14 trials, 6 studies adopted single-arm design, 5 studies were RCT, and 3 were non-RCT.

### Quality assessment

The quality of the methodology and risk of bias of the 15 articles were assessed in accordance with Cochrane RoB2 for randomized trials and ROBINS-I for nonrandomized trials^25,26^ (Table 2 and 3).

**Table 2:** Summary of risk of bias assessment of non-randomized studies by Cochrane’s Risk Of Bias In Non-randomized Studies—of Interventions (ROBINS-I) tool

Study	d1	d2	d3	d4	d5	d6	d7	Overall
Tankou et al.^30,31^	**Moderate**	*Low*	*Low*	*Low*	*Low*	*Low*	**Moderate**	**Moderate**
Barone et al.^29^	**Moderate**	*Low*	*Low*	*Low*	*Low*	***Serious***	**Moderate**	***Serious***
Becker et al.^34^	**Moderate**	*Low*	**Moderate**	*Low*	*Low*	***Serious***	**Moderate**	***Serious***
Hegelmaier et al.^36^	***Serious***	***Serious***	*Low*	*Low*	*Low*	**Moderate**	***Serious***	***Serious***
Rusch et al.^37^	**Moderate**	*Low*	*Low*	*Low*	*Low*	**Moderate**	**Moderate**	**Moderate**
Kountouras et al.^40^	**Moderate**	***Serious***	*Low*	*Low*	*Low*	***Serious***	**Moderate**	***Serious***
Hong et al.^39^	***Serious***	*Low*	**Moderate**	*Low*	**Moderate**	**Moderate**	*Low*	***Serious***
Leblhuber et al.^42^	***Serious***	*Low*	**Moderate**	*Low*	*Low*	*Low*	**Moderate**	***Serious***
Kuai et al.^35^	**Moderate**	*Low*	*Low*	*Low*	*Low*	**Moderate**	*Low*	**Moderate**

d1: Confounding bias, d2: Selection of participants into the study, d3: Bias in classification of intervention, d4: Bias due to deviations from intended interventions, d5: Bias due to missing data, d6: Bias in measurement of outcomes, d7: Bias in selection of the reported result.

D1: Bias arising from the randomizing process, D2: Bias due to deviations from intended interventions, D3: Bias due to missing outcome data, D4: Bias in measurement of the outcome, D5: Bias in selection of the reported result.

**Table 3:** Summary of risk of bias assessment of randomized studies by Version 2 of the Cochrane’s Risk Of Bias (RoB 2) tool.

Study	D1	D2	D3	D4	D5	Total		
Al et al^22^	**Some concerns**	**Some concerns**	*Low*	**Some concerns**	*Low*	**Some concerns**		
Cignarella et al.^32^	**Some concerns**	*Low*	*Low*	**Some concerns**	**Some concerns**	**Some concerns**		
Nagpal et al.^41^	*Low*	*Low*	*Low*	*Low*	**Some concerns**	**Some concerns**		
Di Gioia et al.^33^	*Low*	*Low*	*Low*	*Low*	**Some concerns**	**Some concerns**		
Sun et al.^38^	*Low*	*Low*	**Some concerns**	*Low*	*Low*	**Some concerns**		

d1: Confounding bias, d2: Selection of participants into the study, d3: Bias in classification of intervention, d4: Bias due to deviations from intended interventions, d5: Bias due to missing data, d6: Bias in measurement of outcomes, d7: Bias in selection of the reported result.

D1: Bias arising from the randomizing process, D2: Bias due to deviations from intended interventions, D3: Bias due to missing outcome data, D4: Bias in measurement of the outcome, D5: Bias in selection of the reported result.

Bias in the selection of reports is a common concern among studies; here, three out of five RCTs and seven out of nine non-RCTs were of moderate-to-high risk in the concerning domain owing to multiple measurements of disease progression. In general, RCTs had a lower risk of bias, in which no high-risk RCTs were included. The RCT performed by Al et al. was terminated early because of the sudden death of the principal investigator^22^; nevertheless, results were analyzed in such a way that no directional bias toward or against the intervention exists, and this RCT was therefore assessed to have a moderate risk.

For non-RCTs, 66.78% of the studies were of serious risk of bias, and the remaining were of moderate risk^29,34,36,39,40,42^. Apart from reporting bias (D7), major concerns of bias included confounding bias (D1), selection bias (D2), and bias in data measurement (D6). Serious confounding bias mainly contributed to the lack of control of diet, which can affect the microbiome composition, leading to less conclusive results. In addition, many of the non-RCTs relied on self-reporting, while the participants were aware of the interventions. Becker et al. conducted an open-label study to modify GM in PD patients with resistance starch and collected subject-reported nonmotor data; consequently, the measurement could be inaccurate owing to subjective reporting^34^. Poor selection of participants and missing data were also common among nonrandomized clinical trials.

### MS and amyotrophic lateral sclerosis

Five studies focusing on MS^22,29–32^ and one study specifically on ALS^33^ were included. These studies comprised three RCTs^22,32,33^ and two single-arm studies^29–31^. The sample sizes of MS studies were small, ranging from 9^22^ to 22^30,31^, whereas the study on ALS recruited 50 samples^33^. The study durations varied from 1 week^29^ to 1 year^22^. A spectrum of microbiome-modulating interventions were used, ranging from probiotic supplementation^30,31,33^, dietary intervention (intermittent fasting)^32^, FMT^22^, and a multidimensional program consisting of dietary intervention and physical activities^29^. All studies used 16 s rRNA sequencing to analyze microbiome composition, covering V3 and/ or V4 regions. In addition to V3 and V4 regions, V13 region was also covered by Cignarella et al. to distinguish specific species of *Lactobacillus*, as well as V1 and V2 regions^32^.

MS is characterized with chronic inflammatory response in the central nervous system, which leads to pronounced Th1/Th17-mediated inflammation and increased proinflammatory cytokine concentration^43,44^. Therefore, improvement in MS progression can be evaluated by measuring inflammatory response and clinical functional and nonfunctional outcomes. Three out of four studies focusing on MS reported reduced inflammatory response or improved autoimmune response^29–32^, while the remaining underpowered study showed insignificant difference post-modulation^22^.

All of the studies had diverse microbiome patterns. The two studies on probiotic supplementation showed time-related changes in microbiome composition but had different microbiota outcomes despite using similar bacterial families, that is, Di Gioia et al. used Streptococcaceae and Lactobacillaceae families, while Tankou et al. used Bifidobacteriaceae family in addition to Streptococcaceae and Lactobacillaceae families^30,31,33^. Di Gioia et al. found no significant alterations in microbiota and probiotic supplementation, except for *Rikenellaceae* and trends of increase in *Bateroidaceae* and decrease in *Prevotellaceae* and *Clostridiales*, and no clinical improvement in ALS^33^. Tankou et al. reported enrichment of *Lactobacillaceae*, *Streptococcaceae*, and *Bifidobacteriaceae* and reduction of *Akkermansia*, *Blautia*, and *Dorea*, which were enriched in MS patients at baseline. They also observed reduced intermediate monocytes, increased effector memory CD8 T cells, and anti-inflammatory gene expression, with some association with microbiome changes, therefore suggesting an implication of synergistic effect with current therapies^30,31^.

Dietary interventions significantly improved disease progression and inflammatory tone in both studies^29,32^. Cignarella et al. reported increased *Faecalibacterium, Lachnospiracea incertae sedis*, and *Blautia,* improved Expanded Disability Status Scale (EDSS), and reduced serum leptin and peripheral blood leukocyte profile changes after 15 days of intermittent fasting in conjunction with corticosteroid treatment^32^. However, the difference in improvement of EDSS between the ad libitum control group and the intermittent fasting group was insignificant, and the MS Functional Composite was insignificantly different from the baseline in both groups, possibly due to the short intervention duration^32^. Barone et al. conducted a 1-week multidimensional program involving Mediterranean diet, neuromotor rehabilitation, and mindfulness^29^. They reported partial recovery of gut dysbiosis with reduced *Collinsella*, *Actinobacteria*, and *Ruminococcus* and increased *Bacteroidetes* and some short-chain fatty acid (SCFA) producers. They also observed reduced inflammatory tone and serum lipopolysaccharide, increased anti-inflammatory gene expression, and some associations with microbiome changes. Considering the significant improvement in the total score in the Modified Fatigue Impact Scale, the author concluded that the multidimensional approach may be effective in mitigating MS progression.

Al et al. conducted an RCT on FMT, in which they randomized participants into early (received FMT with 6-month follow-up, n = 4) or late intervention group (6-month observation, substantiated by FMT and 1-month follow-up, n = 5); however, the study was terminated early and underpowered because of the unexpected death of the principal investigator^22^. Preliminary results showed that FMT was well-tolerated without serious adverse events. Microbiome changes recapitulated the microbiome composition of the donor and had the potential to improve elevated intestinal permeability. Insignificant clinical changes were noted on disease severity measured by EDSS, without new MRI activity, and the inflammatory levels in terms of serum cytokines showed insignificant changes.

### PD

We identified six articles that fulfilled all criteria^34–39^. They included one RCT^38^, two non-RCT^34,36^, and three single-arm studies^35,37,39^, which used diversified microbiome-modulating methods, including prebiotics, FMT, dietary interventions, and probiotics. The sample size was in the range of 8–11 for non-RCTs and single-arm studies and 87–100 for RCTs. The study lasted 7 weeks to 1 year.

Two of the studies investigated GM with metagenomic sequencing^34,38^, while others used 16 s rRNA sequencing, despite sequencing different variable regions. Although different interventions were used, the alpha and beta diversities did not differ significantly in all studies. No consensus existed in terms of the change in a particular family, genus, or species.

Despite using different microbiome-modulating strategies, these studies showed a significant impact on alleviating disease burden. Motor functions, as measured by Unified PD Rating Scale (UPDRS), were significantly improved in four of the six studies that used FMT, probiotics, ovo-lacto diet, ovo-lacto diet with enema, and Mediterranean diet^35,36,38,39^, while others did not measure motor function. Apart from the study that used resistant starch^34^, constipation and GI-related symptoms were also improved in the three studies that used probiotics, FMT, and Mediterranean diet^35,37,38^. Other nonmotor symptoms, including anxiety and depression, were improved^34,35,38^, and inflammatory and PD-related fecal markers decreased^34,39^.

Sun et al. provided the only RCT that measured motor, nonmotor, and constipation symptoms, as well as microbiome-related metabolites^38^. Their study evaluated the synergistic effects of probiotics with conventional PD treatment (benserazide and dopamine agonist) by comparing it with a placebo group (placebo with conventional regimen) for 3 months. The probiotics led to increased *Bifidobacterium animalis, Ruminococcaceae*, and *Lachnospira* and decreased *Lactobacillus fermentum* and *Klebsiella oxytoca*, which might be related to changes in microbiome-related metabolites and neurotransmitter, consequently leading to a beneficial effect in PD patients.

### AD

Three included studies focused on AD or mild cognitive impairment, and the study designs were diversified: one single-arm^42^, RCT^41^, and non-RCT^40^. Changes in microbiome were measured by qPCR of designated microbial targets^42^, 16 s rRNA^41^, and histology and urease test of *Helicobacter pyroli*^40^. Given that the focus of the studies varied, they also modified GM with different approaches, including probiotic supplementation^42^, dietary treatment^41^, and antibiotic treatment^40^. The sample sizes ranged from 17^41^ to 61^40^, and the studies lasted for 4 weeks^42^ to 2 years^40^.

Leblhuber et al. investigated the effects of probiotic supplementation on immune activation^42^. They found that 4-week probiotic supplementation led to increased *Faecalibacterium prausnitzii* and altered tryptophan metabolites, yet no significant improvement in cognition was observed. Nagpal et al.^41^ was the only cross-over study to compare the effects of Mediterranean-keto diet (MMKD) on AD markers. They found increased abundance of several bacterial families and genera, such as *Enterobacteriaceae, Akkermansia*, and *Slackia*, after modified MMKD compared with that after American Heart Association Diet (AHAD), which also altered the SCFA profile and was in association with CSF biomarkers, such as Ab40 and Ab42. Kountouras et al. focused on the impact of eradication of *H. pylori* in infected AD patients^45^. AD patients were significantly more susceptible to *H. pylori* infection, and the eradication therapy led to improved cognitive and functional status upon the 2-year clinical endpoint compared with infected patients. These studies suggested that modulating GM may alter AD progression via modifying SCFA and immune profile, leading to reduced AD marker and possibly improved status.

### SCFA producers and fecal/serum SCFAs

Several included studies have explored the relationship between SCFA producers or fecal/serum SCFA concentration in NDDs. Of the eight studies that reported changes in SCFA producers or SCFA concentration, six reported improved outcomes, as measured by inflammatory tone, functional outcome, or GI symptoms, yet showed inconsistent changes in SCFA producers and SCFA concentration (Table 4).

**Table 4:** Summary of studies that measured changes in SCFA producers or SCFA concentration and their clinical outcomes.

Study	Intervention group	SCFA producer/serum/faecal SCFA changes	Clinical outcomes
Tankou et al^30,31^	Probiotic supplementation for two months	decreased *Blautia, Dorea, and B. adolescentis* in both healthy controls and MS patients	⊕ ⊕ ⊕ Decreased inflammatory tones as shown by increased anti-inflammatory gene expression and decreased pro-inflammatory gene expression
Barone et al^29^	1-week High-Impact Multidimensional Rehabilitation	*Blautia* remained stable, and increased *Coprococcus*, *Bacteroides*, and *Oscillospira*, but other SCFA producers reduced	⊕ ⊕ ⊕ Improved MFIS-5, 6MWT, 6MWT-dynamic index Decreased CD4 + /IFN-γ + , Th1, CD4 + /ROR-γ + , CD4 + /IL-17 + , and Th17 and serum LPS. Increased I-FABP
Cignarella et al^32^	Alternate-day fasting for 15 days with corticosteroid treatment	increasing trend of *Faecalibacterium, Lachnospiracea_incertae_sedis and Blautia*	⊕ ⊕ improved EDSS without any significant difference in the degree of amelioration, no difference in MSFC and SDMT **Adipokines and metabolites:** Increased serum adiponectin but no change in beta-hydroxybutyrate, [I]: Decreased BMI to significantly different from [C], Reduced serum leptin
Kuai et al^35^	1-time FMT	*Blautia, Dorea* and *Romboutsia* became dominant Significant and gradual decrease in *Bacteroides* Increased *Faecalibacterium* at 4 and 12 weeks post-FMT, increased *Blautia* at 8 and 12 weeks post-FMT	⊕ ⊕ ⊕ H-Y scores decreased, UPDRS, NMSQ, PAC-QOL, Wexner constipation score increased, small intestinal bacterial overgrowth decreased, HCY expression increased
Rusch et al.^37^	5-week Mediterranean diet	*Roseburea* increased at week 5 *Blautia, Dorea and Lachnospiraceae* decreased	⊕ ⊕ Decreased body weight, significantly lower GSRS constipation and indigestion score, unchanged number of bowel movement and BSS; intestinal permeability did not differ
Sun et al^38^	Daily probiotics for six months plus conventional drugs	increased diversity of SGBs in SCFA synthesis; **SCFAs:** higher serum acetate at T1 and T2. Significantly higher dopamine, and significantly lower glutamine and tryptophan concentration in at T1 in [I] than [C]	⊕ ⊕ ⊕ **Functional/nonfunctional outcomes:** [I]&[C]: improved UPDRSIII, MMSE, HAMA, HAMD-17, more significant improvement in [I]. Improvement in UPDRS-III was only significant at T2 in [C]. [I]: improved PDSS and higher possibility of continuing medication ([C]: received 3-month placebo followed by 3-month probiotics) [I]: improved GI-related symptoms such as times of spontaneous defecation and completed defecation per week
Nagpal, et al.^41^	Modified Mediterranean Keto diet (MMKD)	The abundance of family *Bifidobacteriaceae,* genus *Bifidobacterium* decreased **SCFAs:** slightly reduces faecal lactate and acetate while increasing propionate and butyrate	⊕ No significant correlation found between SCFA producers/SCFA and CSF biomarkers Post-MMKD
Leblhuber et al.^42^	Probiotic supplementation	increased *Faecalibacterium prausnitzii*	⊕ **Functional/nonfunctional outcomes:** cognitive parameters unchanged (MMSE and CDT) **Inflammatory tone:** Decreased faecal zonulin concentrations and increased serum kynurenine and nitrite concentration. Delta values (before—after) of neopterin and the kynurenine to tryptophan ratios (Kyn/Trp) correlated significantly (*p* < 0.05)

Ratings on functional outcome improvement, ⊕ : no significant improvement, ⊕  ⊕ : some degree of improvement, ⊕  ⊕  ⊕ : significant improvement.

Tankou et al. and Rusch et al. reported decreased *Blautia* after probiotic supplementation and Mediterranean diet, respectively^30,31,37^. Barone et al. reported a stable level of *Blautia* after multidimensional rehabilitation^29^. Kuai et al. and Cignarella et al. reported increased *Blautia* after FMT and intermittent fasting^32,35^. All of them, except Cignarella et al., reported improved clinical outcomes. *Faecalibacterium* was reported to have increased significantly in three studies after intermittent fasting, FMT, and probiotic supplementation. Sun et al. reported increased diversity of SGBs involved in SCFA synthesis after probiotic supplementation, in which the acetate and dopamine concentrations increased significantly, whereas the glutamine and tryptophan concentrations decreased^38^. The patients also exhibited improved clinical outcomes.

### Meta-analysis: effect of microbiome modulation on clinical outcomes

**Figure 2:**
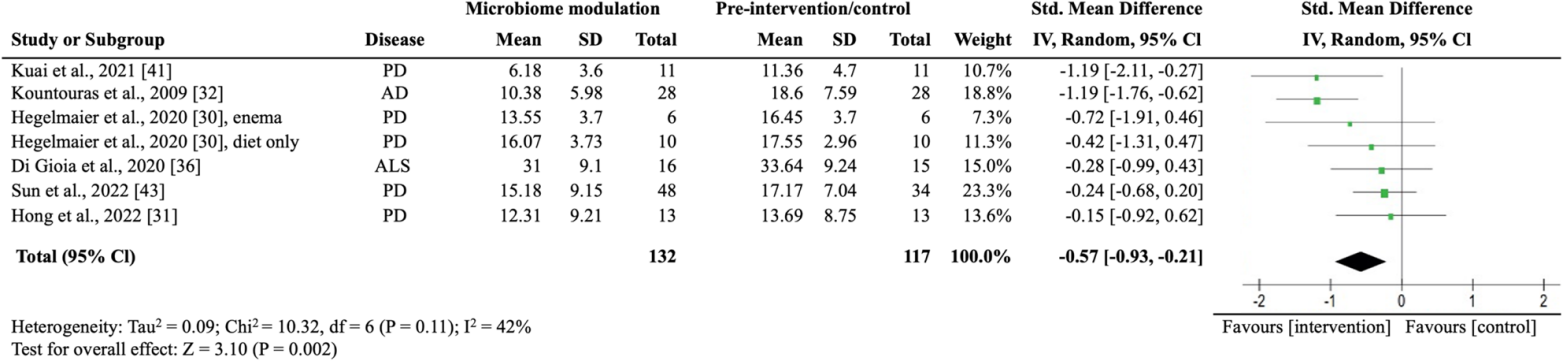
Random-effects meta-analysis of trials on the association between microbiome modulating intervention and clinical outcomes. *AD* Alzheimer’s disease; *ALS* amyotrophic lateral sclerosis; *IV* inverse variance; *PD* Parkinson’s disease; error bars represent 95% CIs; size of the shaded square indicates study weight; diamond represents pooled standardized mean difference and 95% CI.

We pooled all clinical trials to investigate the overall effectiveness of modulating the microbiome on motor symptom progression in NDD. Of the eight studies that assessed motor symptom progression, five (62.5%) reported statistically significantly improvements in motor symptom progression. We pulled all studies that reported motor symptom progression quantitatively before and after intervention and extracted the mean, interquartile range, or SD to calculate the standard mean differences of the studies, resulting in six studies. We did not include Al et al.’s study in meta-analysis, despite the reported EDSS, due to the study’s early termination, which resulted in incomplete and varied treatment conditions in the two groups^22^. Additionally, we excluded the study conducted by Cignarella et al. as it did not report numerical data for EDSS^32^. In the meta-analysis involving six studies with seven intervention groups (n = 249), microbiome-modulating interventions were significantly associated with a lower motor symptom burden (SMD, − 0.57; 95% CI − 0.93 to − 0.21; I^2^ = 42%; *P* = 0.002; Fig. 2). They used different strategies, such as probiotics, antibiotics, FMT, and dietary changes, to alter the gut microbiota^33,35,36,38–40^. Four out of the six studies included patients with PD^30,31,35,36,39^, and the remaining involved patients with ALS^33^ and AD^40^. The primary outcome measures were UPDRS for PD, ALS Functional Rating Scale-Revised for ALS, and Functional Rating Scale for Symptoms of Dementia for AD. Hegelmaier et al. compared the clinical outcomes of PD patients receiving ovo-lacto diet, with a subgroup receiving additional enema^36^. Considering that the aim of this meta-analysis was to study the pooled effect of microbiome-modulating methods, we compared the clinical outcomes before and after interventions and segregate the enema subgroup. We did not include studies on MS because they did not report numerical results on functional outcomes or were underpowered.

### Constipation and GI symptoms

**Figure 3:**
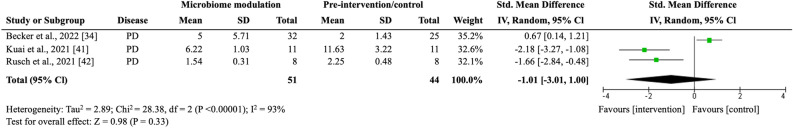
Random-effects meta-analysis of trials on the association between microbiome modulating intervention and constipation symptoms. *IV* inverse variance; *PD* Parkinson’s disease; error bars represent 95% CIs; size of the shaded square indicates study weight; diamond represents pooled standardized mean difference and 95% CI.

Of the four trials that assessed constipation and GI-related symptoms^33–35,37^, three studies reported significant improvement compared with the baseline or placebo, whereas the remaining reported insignificant changes in bowel habits. The meta-analysis did not include Hegelmaier et al.’s study because they used the Bristol stool scale, a noncontinuous scale, in measuring constipation syndrome^36^. In the meta-analysis involving three trials (n = 76), microbiome-modulating interventions were insignificantly associated with improving constipation (SMD, − 1.01; 95% CI − 3.01 to 1.00; I^2^ = 93%; *P* = 0.33; Fig. 3). The primary outcome measures included the Constipation Scoring System^34^, Wexner^35^, and GI Symptom Rating Scale^37^ constipation scores.

## Discussion

This study is the first systematic review and meta-analysis to date synthesizing the current evidence from clinical trials that examined the effects of microbiome-modulating interventions on the disease burden of NDDs. Our meta-analysis demonstrated that microbiome-modulating interventions are significantly associated with reduction in motor symptom burden in NDDs, including PD, ALS, and AD. Findings from qualitative synthesis also suggested that microbiome-modulating interventions may reduce inflammation and alleviate GI symptoms, including constipation. Despite the promising effects of microbiome-modulating interventions, the relationships and mechanisms underpinning GM modulation and clinical outcomes remain inconclusive owing to the lack of high-quality clinical trials, the heterogeneity in study design, and the diverse nature of interventions among the included studies.

### Microbiome modulation may improve motor symptoms and inflammatory tone

Our meta-analysis revealed that microbiome-modulating interventions can generally lower motor symptom burden in patients with NDDs. In addition, qualitative findings showed that inflammatory tone was generally improved in different NDDs by various microbiome-modulating interventions. NDDs are characterized by chronic inflammation, leaky gut, and decreased production of neuroactive substances, in which the degeneration and loss of neurons lead to long-term motor and nonmotor impairment^45,46^. The effect on alleviation on symptom burden might be explained by the restoration of GM to reduce inflammation^47^, re-establish intestinal permeability^22,37,48^, and enhance neuroactivity through the production of neurotransmitters^49^.

Xiang et al. performed systematic review and meta-analysis on the use of probiotics in AD and PD and suggested that probiotics improve AD possibly through anti-inflammatory pathways, as demonstrated by a decrease in the GSH level after probiotic supplementation^50^. In line with our study findings, microbiome modulation, not limited to probiotic supplementation, was found to reduce inflammation and thereby disease burden, which also applies to other NDDs including MS and ALS^22,29–33,39^.

Restoration of gut dysbiosis can reduce inflammation by multiple pathways, with many of the modulation methods focusing on increasing SCFA-producing bacteria, such as *Roseburia* spp., *Blautia*, and *Prevotella* spp., to increase serum or fecal SCFAs^30,31,34,38,42^ or on reducing pathogenic bacteria, such as *H. pyroli*^39,40^. SCFAs, including butyrate, propionate, and acetate, exert anti-inflammatory effects by inhibiting the activation of nuclear factor-kappa B and the production of proinflammatory cytokines, such as tumor necrosis factor alpha and interleukin-6^51,52^. They can also promote the differentiation of regulatory T cells (Tregs) and suppress that of Th17 cells^53^. Apart from indirect homeostasis through SCFAs, some bacteria in the microbiome, such as *Bacteroides fragilis*, can directly induce Treg differentiation to maintain immune intolerance and prevent autoimmunity.

Along with consistent findings of decreased *Lactobacillus* in MS, Tankou et al. reported an enrichment of SCFA producers, including *Akkermansia*, *Blautia*, and *Dorea*, in MS patients at baseline*.* After probiotic supplementation, these SCFA producers decreased, but the expression of proinflammatory genes, such as HLA.DPA1 and MS risk allele HLA.DQA1, also decreased^30,31^. Our qualitative finding also showed that no consistency was established in the changes in SCFA producers or SCFA concentration, but clinical outcomes were improved in general (Table 4). In particular, while some studies suggested that certain species of *Blautia and Dorea* were associated with decreased levels of inflammatory markers^54,55^, others indicated that they had proinflammatory effects^56,57^. The activity of SCFA producers can vary depending on several factors and contribute differently in terms of SCFA production in the gut^58,59^. Given the complex nature of GM, additional studies are needed to elucidate other factors that influence its interactions with the immune system, such as its abundance, diversity, metabolites, or co-occurrence with other bacteria.

### Microbiome modulation may alleviate constipation and GI symptoms

Constipation is common in NDDs and can affect the quality of life of patients. Constipation can be caused by the accumulation of pathological proteins in the GI tract, such as amyloid beta in AD, α-synuclein in PD, or myelin basic protein in MS, which induce dysfunction of the enteric nervous system (ENS) to affect gut motility and barrier^59,60^. Our study found that, qualitatively, microbiome-modulating interventions may also alleviate constipation. A reduction in constipation might be explained by the enhancement in the integrity and permeability of the intestinal barrier through restoring the microbiome^61,62^. In addition, SCFAs produced by bacteria can modulate intestinal peristalsis and upregulate the expression of tight junction proteins to strengthen the integrity of the gut barrier^63^. Secretion of neurotransmitters to stimulate ENS may also play a role in constipation^64^. However, available evidence remains inadequate, and the results have not reached statistical significance because of the lack of high-quality studies, which should ideally be blinded RCTs with appropriate sample size and statistical power.

### Microbiome-modulating interventions and related GM changes

Although all of the included studies reported some degree of changes in GM composition, no consistent changes in GM were found in relation to the overall improvement in clinical outcomes. The inconsistency might be explained by the heterogeneity in interventions and disease nature, while other systematic reviews also observed diverging GM patterns^65,66^. In terms of studies that involved the use of probiotics, *Lactobacillus* and *Bifidobacterium* were commonly used, yet they resulted in different GM changes: one reported increased *Lactobacillus and Bifidobacterium*^30,31^, one reported increased *B. animalis* but decreased *Lactobacillus fermentum*^38^, and one reported an increase in *F. prausnitzii* only^42^. These observations are in line with other probiotic systematic reviews^67^. The exact relationship between GM and NDDs remains unknown, and further studies are needed to understand the impact of individual bacteria, the co-occurrence, and the molecular pathway in GM and diseases.

### Strengths and limitations

This is the first comprehensive systematic review that examined the effects of a broad spectrum of microbiome-modulating interventions on NDDs, including MS, ALS, PD, and AD. NDDs represent a broad spectrum of disorders, and clinical microbiome trials remain lacking for some diseases, such as the Huntington disease. In addition, most included studies could only be synthesized qualitatively, and heterogeneity regarding the intervention type, outcome measures, and methodological differences was noted. Therefore, we adopted a random-effect model to account for the statistical heterogeneity among studies. Publication bias assessment was not possible given the limited number of available trials for quantitative synthesis, which may also result in minimal but statistically significant overestimation of effects^68^. Most of the included clinical trials had a small sample size and were of moderate risk of bias mainly subject to the selection of reported results. We also included nonrandomized and single-arm clinical trials, which might have a high risk of bias owing to the lack of comparison group. Studies should include a control group when possible and report the complete effect estimate on the basis of the *P* value, magnitude, or direction of results^69^, such as fold change in microbiome changes. When evaluating microbiome diversity, using multiple indices can provide a comprehensive and nuanced understanding of the microbiota diversity and composition^70^, yet all results of the chosen indices should be listed and interpreted to prevent reporting bias. The findings indicate that the relationship between microbiome-modulating interventions, GM composition, and clinical outcomes of NDDs has been poorly studied and skewed to certain NDDs, namely, PD.

A spectrum of microbiome-modulating components was identified, ranging from probiotic supplement to multidimensional lifestyle interventions consisting of diet modification, mindfulness, and physical activities. Owing to the large variation in methodology across the included studies, definitive conclusions on how microbiome-modulating interventions modulate the GM composition and clinical outcomes and affect the progression of NDDs were impossible to draw.

Most of the studies did not report on significant confounders, such as comorbidities, medication use, and lifestyle, which could affect microbial and clinical outcomes and thus might limit the transferability of our findings. Control conditions also differed between the studies, given that some control interventions comprised AHAD^41^, a placebo group that received conventional treatments^32,38^, or a group that received placebo for the first 3 months and probiotics for the next 3 months^33^, restricting the generalizability of our study findings.

## Conclusions and implications

Microbiome-modulating interventions are likely to improve symptom burden, possibly through reducing inflammatory tone in NDD patients via increasing SCFA producers and reducing proinflammatory bacteria. However, the exact relationship remains unknown because no consistent changes in GM composition were identified. High-quality evidence of microbiome-modulating interventions for NDDs is still missing. This review underscores the need for rigorous large-scale studies to examine the effects of microbiome-modulating methods on NDDs.

Future clinical trials of microbiome-modulating methods on NDDs should (1) evaluate the changes in GM through microbiome modulation in terms of alpha and beta diversities and specific phylum, family, and species; (2) assess motor and nonmotor clinical outcomes and incorporate objective data in addition to self-reporting questionnaire; (3) account for confounding factors, including diet, age, medication record, lifestyle, and disease progression. Regarding the diverse methodology of existing GM modulation research, a standardized approach to GM evaluation, such as the STORMS checklist^71^, is necessary to understand the complex mechanisms and relationships between GM-modulating interventions, GM composition, and NDDs further.

### Supplementary Information

Supplementary Information.

## Data Availability

The dataset analyzed in this study is available from the corresponding author upon reasonable request.
