# Supplementary material for: Effects of microbiome-based interventions on neurodegenerative diseases: a systematic review and meta-analysis
Source: Sci Rep. 2024 Apr 26;14:9558. doi: 10.1038/s41598-024-59250-w (PMC11045862; doi:10.1038/s41598-024-59250-w)
Supplement: Supplementary file 1 — Supplementary Information. [file 41598_2024_59250_MOESM1_ESM.docx]

**Supplementary Information**

**Manuscript title:**

Effects of microbiome-based interventions on neurodegenerative diseases: A systematic review and meta-analysis

**Journal name:**

Scientific Reports

**Author list:**

1. Zara, Siu Wa CHUI^a^, BBioMed Student
2. Lily, Man Lee CHAN^b^ , PhD Candidate, BN (Hons), RN
3. Esther, Wan Hei ZHANG^c^, MBBS Student
4. Suisha LIANG^d^, PhD Candidate, MEng(BE), BEng(BME)
5. Edmond, Pui Hang CHOI^b^, Assistant Professor, PhD, BN, RN
6. Kris, Yuet Wan LOK^b^, Assistant Professor, PhD, MSc (DIC), BSc (SRD)
7. Hein Min TUN^e,f^, Associate Professor, PhD, MSc, BVSc
8. Jojo, Yan Yan KWOK^b*^, Assistant Professor, PhD, MPH, BN (Hons), RN
   Email: [jojo.yykwok@gmail.com](mailto:jojo.yykwok@gmail.com); [jojoyyk@hku.hk](mailto:jojoyyk@hku.hk)
   ORCID: 0000-0001-7444-6935

^a^ School of Biomedical Sciences, Li Ka Shing Faculty of Medicine, The University of Hong Kong, Pokfulam, Hong Kong SAR

^b^ School of Nursing, Li Ka Shing Faculty of Medicine, The University of Hong Kong, Pokfulam, Hong Kong SAR

^c^ Faculty of Medicine, The Chinese University of Hong Kong, Shatin, Hong Kong SAR

^d^ HKU-Pasteur Research Pole, School of Public Health, Li Ka Shing Faculty of Medicine, The University of Hong Kong, Hong Kong SAR

^e^ The Jockey Club School of Public Health and Primary Care, Li Ka Shing Institute of Health Sciences, The Chinese University of Hong Kong, Shatin, Hong Kong SAR

^f^Microbiota-I Center (MagIC), Hong Kong SAR, China

**Corresponding Author:**

Name: Jojo Yan Yan Kwok

Address: School of Nursing, Li Ka Shing Faculty of Medicine, The University of Hong Kong, Hong Kong SAR

Phone number: (852) 39176644

E-mail address: [jojo.yykwok@gmail.com](mailto:jojo.yykwok@gmail.com); [jojoyyk@hku.hk](mailto:jojoyyk@hku.hk)

**Online Resource 1.** The full Search strategy

PubMed

| Search | Query |
| --- | --- |
| 1 | (Alzheimer*) or (Huntington*) or (Parkinson*) or (multiple sclerosis) or (Amyotrophic lateral sclerosis) or (Neurodegenerative disease) or ([Spinal muscular atrophy](https://medlineplus.gov/spinalmuscularatrophy.html)) or ([Lewy body disease](https://medlineplus.gov/lewybodydementia.html)) or (Friedreich ataxia) |
| 2 | (bacter*) or (dysbiosis) or(microbiota) or (gastrointestinal microbiome) or (microbiome analy?ing technique) or (16s rRNA) or (shotgun sequencing) or (FISH) or (microflora) or (microbial) or (gut) or (microbiome) or (gut flora) or (gastrointestinal metagenome) |
| 3 | (diet therapy) or (vegan) or (carbohydrate-restricted) or (fat-restricted) or (caloric restriction) or (western diet) or (diet) or (keto*) or (Mediterranean diet) or (low fat) or (low calorie) or (intermittent fasting) |
| 4 | (dietary supplement) or (vitamin*) or (omega) or (prebiotics) or (probiotics) or (postbiotics) or (symbiotics) or (antibiotics) |
| 5 | (faecal microbiota transplant) or (stool transplant) or (fecal bacteriotherapy) or (fecal transfusion) or (bacteriotherapy) or (intestinal microbiota transplant) |
| 6 | #1 AND #2 AND (#3 OR #4 OR #5) |

Web of Science

| Search | Query |
| --- | --- |
| 1 | (Alzheimer*) or (Huntington*) or (Parkinson*) or (multiple sclerosis) or (Amyotrophic lateral sclerosis) or (Neurodegenerative disease) or ([Spinal muscular atrophy](https://medlineplus.gov/spinalmuscularatrophy.html)) or ([Lewy body disease](https://medlineplus.gov/lewybodydementia.html)) or (Friedreich ataxia) |
| 2 | (bacter*) or (dysbiosis) or(microbiota) or (gastrointestinal microbiome) or (microbiome analy?ing technique) or (16s rRNA) or (shotgun sequencing) or (FISH) or (microflora) or (microbial) or (gut) or (microbiome) or (gut flora) or (gastrointestinal metagenome) |
| 3 | (diet therapy) or (vegan) or (carbohydrate-restricted) or (fat-restricted) or (caloric restriction) or (western diet) or (diet) or (keto*) or (Mediterranean diet) or (low fat) or (low calorie) or (intermittent fasting) |
| 4 | (dietary supplement) or (vitamin*) or (omega) or (prebiotics) or (probiotics) or (postbiotics) or (symbiotics) or (antibiotics) |
| 5 | (faecal microbiota transplant) or (stool transplant) or (fecal bacteriotherapy) or (fecal transfusion) or (bacteriotherapy) or (intestinal microbiota transplant) |
| 6 | #1 AND #2 AND (#3 OR #4 OR #5) |

Ovid Embase

| Search | Query |
| --- | --- |
| 1 | (Alzheimer*) or (Huntington*) or (Parkinson*) or (multiple sclerosis) or (Amyotrophic lateral sclerosis) or (Neurodegenerative disease) or ([Spinal muscular atrophy](https://medlineplus.gov/spinalmuscularatrophy.html)) or ([Lewy body disease](https://medlineplus.gov/lewybodydementia.html)) or (Friedreich ataxia).ab,ti |
| 2 | (bacter*) or (dysbiosis) or(microbiota) or (gastrointestinal microbiome) or (microbiome analy?ing technique) or (16s rRNA) or (shotgun sequencing) or (FISH) or (microflora) or (microbial) or (gut) or (microbiome) or (gut flora) or (gastrointestinal metagenome).ab,ti |
| 3 | (diet therapy) or (vegan) or (carbohydrate-restricted) or (fat-restricted) or (caloric restriction) or (western diet) or (diet) or (keto*) or (Mediterranean diet) or (low fat) or (low calorie) or (intermittent fasting).ab,ti |
| 4 | (dietary supplement) or (vitamin*) or (omega) or (prebiotics) or (probiotics) or (postbiotics) or (symbiotics) or (antibiotics).ab,ti |
| 5 | (faecal microbiota transplant) or (stool transplant) or (fecal bacteriotherapy) or (fecal transfusion) or (bacteriotherapy) or (intestinal microbiota transplant).ab,ti |
| 6 | #1 AND #2 AND (#3 OR #4 OR #5) |

**Online Resource 2.** Study Eligibility Criteria

**Inclusion criteria:**

- Clinical Trial (Randomized AND non-randomized AND pilot study)
- Study a type of neurodegenerative disease: Alzheimer’s’, Parkinson’s, Amyotrophic Lateral sclerosis, Multiple Sclerosis, etc.
- Involve interventions that aim to modify the gut microbiome, including but not limited to dietary interventions, probiotics, prebiotics, synbiotics, antibiotics, faecal microbiome transplant, and behavioural therapy
- Performed microbiome analysis, including but not limited to 16s rRNA, whole genome sequencing, shotgun sequencing, and fluorescence in-situ hybridization
- Evaluate neurodegenerative disease progression / disease symptoms (e.g. cognitive and psychological functions)
- Publication in English

**Exclusion Criteria:**

- Participant age <18
